# Is the Concept of Quality of Life Relevant for Multiple Sclerosis Patients with Cognitive Impairment? Preliminary Results of a Cross-Sectional Study

**DOI:** 10.1371/journal.pone.0030627

**Published:** 2012-01-23

**Authors:** Karine Baumstarck, Jean Pelletier, Valérie Aghababian, Françoise Reuter, Irina Klemina, Julie Berbis, Anderson Loundou, Pascal Auquier

**Affiliations:** 1 EA3279 Self-Perceived Health Assessment Research Unit and Department of Public Health, Nord University Hospital, APHM, Marseille, France; 2 Departments of Neurology and CRMBM CNRS6612, Timone University Hospital, APHM, Marseille, France; 3 EA 3273 Psychology of Cognition, Language, and Emotion Research Centre, Aix-Marseille University, Aix-en-Provence, France; University of British Columbia, Canada

## Abstract

**Background:**

Cognitive impairment occurs in about 50% of multiple sclerosis (MS) patients, and the use of self-reported outcomes for evaluating treatment and managing care among subjects with cognitive dysfunction has been questioned. The aim of this study was to provide new evidence about the suitability of self-reported outcomes for use in this specific population by exploring the internal structure, reliability and external validity of a specific quality of life (QoL) instrument, the Multiple Sclerosis International Quality of Life questionnaire (MusiQoL).

**Methods:**

*Design:* cross-sectional study. *Inclusion criteria*: MS patients of any disease subtype. *Data collection*: sociodemographic (age, gender, marital status, education level, and occupational activity) and clinical data (MS subtype, Expanded Disability Status Scale, disease duration); QoL (MusiQoL and SF36); and neuropsychological performance (Stroop color-word test). *Statistical analysis*: confirmatory factor analysis, item-dimension correlations, Cronbach's alpha coefficients, Rasch statistics, relationships between MusiQoL dimensions and other parameters.

**Principal Findings:**

One hundred and twenty-four consecutive patients were enrolled. QoL scores did not differ between the 69 cognitively non-impaired patients and the 55 cognitively impaired patients, except for the symptoms dimension. The confirmatory factor analysis performed among the impaired subjects showed that the structure of the questionnaire matched with the initial structure of the MusiQoL. The unidimensionality of the MusiQoL dimensions was preserved, and the internal validity indices were satisfactory and close to those of the reference population.

**Conclusions/Significance:**

Our study suggests that executive dysfunction did not compromise the reliability and the validity of the self-reported QoL questionnaires.

## Introduction

Measures of health-related quality of life (HRQoL) are being used with increasing frequency in the treatment of multiple sclerosis (MS) as an outcome measure for assessing disease progression, evaluating treatment and managing care [1], [2]. While regulatory authorities and clinicians request this type of information, HRQoL remains rarely used in clinical practice to adjust the management of the patient care because assessment of HRQoL is suspected of containing some limitations [3].

The use of self-reported outcomes among subjects with cognitive dysfunction is of particular concern [3]. While cognitive impairment occurs in about 50% of MS patients [4], [5], even during the early stages of the disease [6], [7], the extent to which MS patients with cognitive dysfunction can validly self-report their quality of life (QoL) is a crucial issue that remains insufficiently examined. The main argument against using self-reported QoL information from patients with cognitive dysfunction was based on the fact that the QoL instruments were not developed among these specific individuals. Although there is a little evidence concerning the reliability and validity of health status measures in cognitively impaired patients [3], two perspectives have been presented. While some authors have argued that individuals with cognitive impairment are not able to produce valid QoL measures [4], [8], others reported some empirical evidence suggesting that individuals with a moderate degree of cognitive impairment can perform reliable HRQoL assessments [9]–[11]. Most of the studies provided information about patients with severe mental disorders [12]–[15] or older populations [16] presenting with dementia or other severe cognitive impairment [16]–[18]. To our knowledge, only two main studies have reported data from MS patients [10], [11]. These results suggested that cognitive decline does not compromise the reliable and valid assessment of self-reported health measures. These studies did not report how the factorial structure described in the impaired samples fit with the initial structure of the tested instrument, which is a key point when considering validity in these specific populations.

To provide new evidence about the suitability for using self-reported QoL information in this specific population, we propose to explore the internal structure, reliability and external validity of a specific QoL instrument, the Multiple Sclerosis International Quality of Life questionnaire (MusiQoL), exclusively developed from the patients' point-of-view [19]. The study sample includes MS subjects with or without cognitive impairment. The MusiQoL is a self-administered, disease-specific QoL instrument that is available in 14 languages [20]–[23].

## Methods

This study relied on a cross-sectional design and was performed in the neurology department of a French public academic teaching hospital (Marseille, France). The inclusion criteria were as follows: patient with MS diagnosis according to McDonald criteria [24], any disease subtype, no history of psychiatric or neurological disease (other than MS), no history of alcohol/drug abuse, and native French speaker. The French Ethics Committee (Comité de Protection des Personnes Marseille II) approved the study, and patients gave their informed consent to participate. Sociodemographic (age, gender, marital status, education level, and occupational activity) and clinical (MS subtype and disease duration) data for each patient were recorded. The MS disability was assessed using the Expanded Disability Status Scale (EDSS).

HRQoL was assessed by means of the MusiQoL. The MusiQoL is a well-validated questionnaire that describes the following nine dimensions and yields a global index score: activity of daily living (ADL), psychological well-being (PWB), symptoms (SPT), relationships with friends (RFr), relationships with family (RFa), relationships with health care system (RHCS), sentimental and sexual life (SSL), coping (COP), and rejection (REJ). HRQoL assessment was completed using the Short Form 36 (SF36), which is a generic questionnaire [25] describing eight subscales (physical function, social functioning, role physical, role emotional, mental health, vitality, bodily pain, and general health). Two composite scores (physical and mental, PCS-SF36 and MCS-SF36) were also calculated.

Neuropsychological performance was assessed using the carded-based version of the Stroop color-word test [26]. We used the more widespread version including 3 subtests: 1) the color naming subtest where the subject was instructed to name the color of a string of dots; 2) the word naming subtest where the subject was instructed to read a list of words indicating colors printed in black letters; and 3) the color-word naming subtest where the subject had to name the color of the letters of color words printed in different colors. Performance was assessed by calculating the time required to name 100 items in each trial (higher scores indicate worse performance). The test was administered in a standardized manner by the same psychologist (FR) who was intensively trained in test administration. The same instructions were given to the subjects prior to each trial.

For each subtest, the subject was defined as impaired or non-impaired by applying French normative values [27] according to age and educational level. Patients were categorized into the following categories according to cognitive function as measured by the Stroop test: a) cognitively non-impaired group (3 normal subtests); b) cognitively impaired group (one or more abnormal subtests).

### Statistical analyses

**Figure 1:**
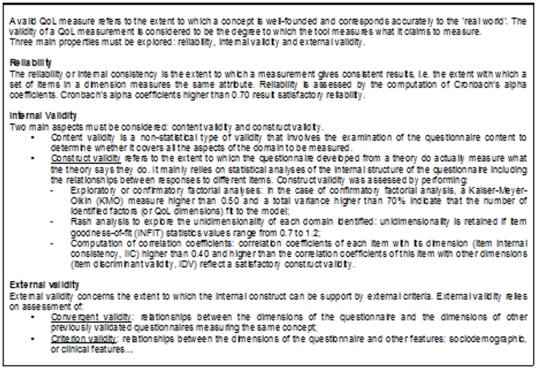
Psychometric properties of a QoL questionnaire: definitions.

Statistical analyses were performed to explore the internal structure, reliability and external validity of the MusiQoL. The exploration of the psychometric properties of a questionnaire was largely described in the specific literature. The definitions of the main psychometric properties were summarized in the Figure 1. Statistical analyses were performed on the two groups defined above using the same procedure reported in the initial validation publication (reference population), except for factor analysis (confirmatory instead exploratory).

The structures of the MusiQoL, both in the non-impaired and impaired groups, were explored using confirmatory factor analysis to determine how these structures matched with the initial structure of the MusiQoL issued of a principal component factor analyses with varimax rotation. Kaiser-Meyer-Olkin (KMO) measures of sample adequacy of the model for the residual matrices were computed: if the KMO index was higher than 0.50, then a factor analysis of the residual matrix was performed.

The multidimensional structure (construct validity) of the version was checked using the multi-trait/multi-item analysis program [28]. Internal structural validity was assessed by investigating item-dimension correlations. Item internal consistency (IIC) was assessed by correlating each item with its scale, and item discriminant validity (IDV) was assessed by determining the extent to which items correlated with the dimension they were hypothesized to represent as compared to correlations with other dimensions. Floor and ceiling effects were reported to assess the homogeneous repartition of the response distribution (effects lower than 10% are expected). For each dimension, internal consistency reliability was evaluated by Cronbach's alpha coefficient [29]; the values of which were compared between the non-impaired and impaired groups using the alpha test program [30].

The unidimensionality of each scale was explored by computation of item goodness-of-fit statistics (INFIT) issued from Rasch analyses [31]. INFIT values ranging from 0.7 to 1.2 ensure that all the items of the scale tend to measure the same concept. Differential item functioning (DIF) analyses were performed, comparing the item difficulties between the two groups according to the cognitive status (non-impaired, impaired) to check whether all the items behave the same way [31]. DIF means that an item performs and measures differently for one subgroup of a population than for another.

To explore external validity, Spearman's correlation coefficients were used to investigate relationships between dimensions of the MusiQoL and SF36 in each group, and the associations between the MusiQoL dimension scores and sociodemographic and clinical features were reported. For qualitative variables, mean dimension scores of the MusiQoL were compared across patient groups that were expected to differ (e.g., gender, educational level, marital status, and occupational status) using one-way analysis of variance. Quantitative variables (e.g., age, EDSS score, and MS duration) were analyzed using Spearman's correlation coefficients. The underlying assumption was that the strength of the relationships would be similar for both groups (non-impaired and impaired) and the reference population. Comparisons of correlation coefficients were performed [32].

Acceptability was assessed by calculating the percentage of missing data per dimension.

Data analyses were performed using SPSS 11.0, MAP-R, LISREL and WINSTEP software.

## Results

One hundred and twenty-four consecutive patients were enrolled. The mean age was 45 years (SD 11), 57.3% of the patients were women, and 47.2% had more than 12 years of education. The MS subtypes included 61 secondary progressive, 36 relapsing remitting, 20 primary progressive, and 7 clinically isolated syndromes. From the French normative values [27], performances on Stroop subtests varied from 24 to 28% (24.1% impaired for the color naming subtest, 26.5% for the word naming subtest, and 28.0% for the color-word naming subtest). The definition of cognitive status classified 69 patients as cognitively non-impaired and 55 (44.3%, 95% confidence interval 35.6–53.0) as cognitively impaired.

### MusiQoL scores

**Figure 2:**
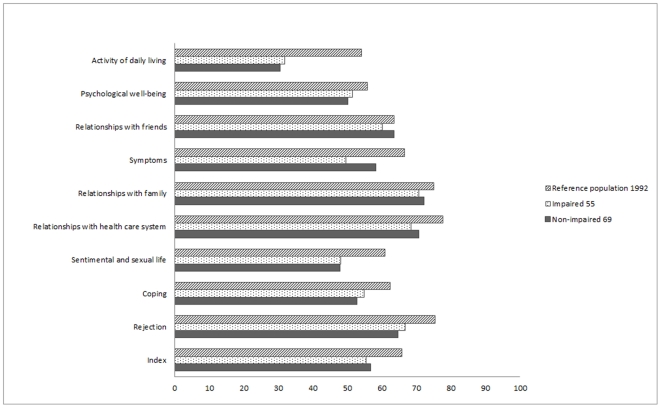
Means of dimension/index scores of MusiQoL according to the cognitive status.

The mean dimension scores and indices did not differ between the non-impaired and impaired subjects except for the symptoms dimension, with higher scores among the non-impaired subjects (Figure 2). Missing values were higher in the impaired group but never exceeded 10% (range from 4.8 to 10.0%). Details are presented in table 1.

**Table 1:** Internal structural validity/reliability/unidimensionality.

	IIC^1^ Min-Max			IDV^2^ Min-Max			Floor %			Ceiling %			Alpha^3^			INFIT^4^			Missing values %		
	NI 69	I 55	*Ref 1992*	NI 69	I 55	*Ref 1992*	NI 69	I 55	*Ref 1992*	NI 69	I 55	*Ref 1992*	NI 69	I 55	*Ref 1992*	NI 69	I 55	*Ref 1992*	NI 69	I 55	*Ref 1992*
ADL	0,42–0,71	**0,40**–0,76	*0.66–0.81*	−0,38–0,41	−0,25–**0,50**	*0,02–0,49*	3,1	2	*1,3*	0	0	*4,6*	0,83	0,86	*0,92*	0,72–**1,56**	**0,59**–**1,74**	*0,86–1,2*	1,4	5,9	*1,4*
PWB	0,57–0,78	**0,61**–0,87	*0,67–0,76*	−0,13–0,48	0,01–**0,66**	*0,09–0,41*	3,1	2	*2,4*	0	2	*4,6*	0,83	0,87	*0,85*	0,78–1,13	**0,52**–1,28	*0,81–1,13*	0,7	6,4	*0,9*
RFr	0,73–0,82	0,70–0,80	*0,69–0,78*	−0,36–0,4	−0,04–0,38	*0,04–0,36*	1,6	2	*2,4*	9,4	8	*13*	0,87	0,87	*0,75*	**0,65**–1,15	0,78–1,26	*0,84–1,15*	0,5	5,5	*7,4*
SPT	0,48–0,61	0,38–0,63	*0,48–0,65*	−0,28–0,27	−0,12–0,35	*0,06–0,41*	0	2	*0,7*	4,7	2	*10,3*	0,76	0,70	*0,80*	0,82–1,17	0,78–1,19	*0,75–1,17*	0,7	5,5	*0,7*
RFa	0,64–0,67	0,58–0,73	*0,62–0,68*	−0,45–0,35	−0,15–0,45	*0,04–0,38*	0	0	*0,8*	20,3	22	*25,7*	0,81	0,80	*0,86*	0,93–1,07	0,76–1,08	*0,88–1,07*	0,5	4,8	*2,3*
RHCS	0,54–0,64	0,41–0,58	*0,42–0,56*	−0,30–0,20	−0,23–0,33	*0,05–0,32*	0	0	*0,3*	14,1	6	*24,5*	0,75	**0,66**	*0,68*	0,78–1,18	0,81–1,14	*0,83–1,18*	0,5	5,5	*2,6*
SSL	0,56–0,56	0,72–0,72	*0,75–0,75*	−0,11–0,25	−0,12–0,43	*0,15–0,43*	18,8	22	*7,6*	9,4	14	*18,7*	0,72	0,84	*0,85*	0,99–1	0,94–1,02	*0,98–1*	7,2	10	*18,8*
COP	0,46–0,46	0,47–0,47	*0,66–0,66*	−0,12–0,44	−0,20–0,41	*0,12–0,45*	6,3	8	*5,8*	6,3	14	*21,1*	**0,63**	**0,64**	*0,80*	0,97–1	0,99–0,99	*0,97–1*	0	5,5	*5,1*
REJ	0,79–0,79	0,82–0,82	*0,60–0,60*	−0,24–0,36	0,04–0,66	*0,13–0,41*	6,3	10	*1,5*	25	44	*32,9*	0,88	0,90	*0,74*	0,97–1,04	0,95–0,96	*0,95–1,04*	0	5,5	*9*

ADL activity of daily living, PWB psychological well-being, RFr relationships with friends, SPT symptoms, RFa relationships with family, RHCS relationships with health care system, SSL sentimental and sexual life, COP coping, REJ rejection.

NI non-impaired, I impaired, Ref reference population.

1 Item-Internal Consistency,

2 Item Discriminant Validity,

3 Cronbach's alpha,

4 Rasch statistics.

Bold values: unsatisfactory values.

Italic characters: reference population values.

### Construct validity

The 9-factor structure of the MusiQoL accounted for 73.4% of the total variance among the non-impaired patients and for 77.3% among the impaired patients.

In the non-impaired group, the 9-factor structure was clearly retrieved. Only 3 of the 31 items contributed to a second factor without being major contributors. In the impaired group, only 8 of the 9 initial factors were identified. Two items (numbers 28 and 29) that belonged to the rejection dimension in the initial structure contributed to another factor: the psychological well-being dimension, which is close to the rejection dimension. All other items mainly contributed to their initial dimension, except item number 15, which was initially caught by the symptoms dimension. The content analysis of the new isolated factor (factor 4) did not identify a specific meaning, grouping both psychological well-being and rejection dimensions. These structures appear acceptable and are presented in the table S1.

Internal structural validity was satisfactory for all dimensions in the two groups; each item achieved the 0.40 standard for IIC. The correlation for each item with its contributive dimension was higher than with the others (IDV), except for two dimensions (i.e., activity of daily living and psychological well-being) in the impaired group. Floor effects were less than 10%, except in the sentimental and sexual life dimension (18.8% among non-impaired subjects and 22.0% among impaired, respectively). The wrong ceiling effects were produced for the rejection dimension, 25.0 and 44.0% respectively. Cronbach's alpha coefficients ranged from 0.63 to 0.88 in the non-impaired group, and from 0.64 to 0.90 in the impaired group, indicating satisfactory internal consistency. No statistical differences were found between the non-impaired and impaired groups using Cronbach's alpha. For 6 of the 9 dimensions, no items showed an INFIT statistic outside the acceptable range; items were outside the acceptable range for activity of daily living in both groups, for relationships with friends in the non-impaired group, and for psychological well-being in the impaired group. All results are detailed in table 1. According to the definition of DIF, there should be no association between the item and the cognitive status, showing that MusiQoL dimensions are relevant whatever the cognitive status (this was the case only for item number 15 with p<0.05, data not shown).

### External and discriminant validity

Spearman's correlation coefficients between MusiQoL and SF36 scores are provided in the table S2. The concepts covered by the MusiQoL and the SF36 are not strictly overlapping. The social functioning domain did not correlate with ‘relationships-like’ dimensions of MusiQoL. As expected, the mental health dimension and mental composite score of the SF36 were mainly statistically associated with the psychological well-being dimension of the MusiQoL, while physical functioning, vitality, bodily pain, general health and physical composite score of SF36 correlated more strongly with the activity of daily living dimension of the MusiQoL, across all cognitive groups. Among the 100 tested correlations, only 80% of them were not statistically different between the non-impaired and impaired groups (results not reported). As expected, few significant correlations were found between MusiQoL scores and MS duration or EDSS, except for EDSS, which highly correlated with activity of daily living in the non-impaired group. Contrarily, the age of patients was not linked to activity of daily living. These results are detailed in table 2. As expected, the women in this study reported lower psychological well-being scores than the men, and single subjects reported lower sentimental and sexual life and index scores than subjects having a partner among both non-impaired and impaired individuals (table 3).

**Table 2:** Correlations between MusiQoL, age and clinical features according to the cognitive status.

		ADL	PWB	RFr	SPT	RFa	RHCS	SSL	COP	REJ	index
Age	NI	−0,04	0,21	0,14	0,01	−0,10	0,10	−0,07	**0,26** *	**0,28** *	0,18
	I	−0,07	0,02	0,06	0,20	0,08	0,08	−0,07	0,08	−0,06	0,08
	*Ref*	***−0,33*** **	*−0,01*	*0,01*	***−0,14*** **	*−0,03*	*0,00*	***−0,13*** **	*0,00*	***−0,05*** *	***−0,13*** **
EDSS	NI	**−0,56** **	0,12	0,23	0,01	0,00	−0,17	−0,06	−0,04	0,19	0,05
	I	−0,16	0,04	0,14	0,26	0,13	−0,08	0,08	0,11	0,12	0,15
	*Ref*	***−0,65*** **	*−0,04*	*−0,03*	***−0,19*** **	*−0,01*	***−0,11*** **	***−0,19*** **	***−0,13*** **	***−0,25*** **	***−0,32*** **
MS duration	NI	−0,13	−0,18	0,09	−0,16	**−0,33** **	−0,07	−0,16	0,04	0,09	−0,17
	I	0,14	0,12	0,10	**0,29** *	0,08	0,13	0,16	−0,01	0,12	**0,29** *
	*Ref*	*−0,02*	*0,01*	*0,03*	***−0,07*** **	*−0,05*	*0,00*	*−0,05*	*0,00*	***0,07*** **	*−0,04*

ADL activity of daily living, PWB psychological well-being, RFr relationships with friends, SPT symptoms, RFa relationships with family, RHCS relationships with health care system, SSL sentimental and sexual life, COP coping, REJ rejection.

NI non-impaired, I impaired, Ref reference population.

Spearman rank correlation coefficients were presented.

Bold values: p<0,05,

*p-value<0,05,

**p-value<0,01.

Italic characters: reference population values.

**Table 3:** Associations between MusiQoL dimension scores and sociodemographic characteristics according to the cognitive status.

		Gender			Educational level			Marital status			Occupational status		
		Women	Men	p	Low	High	p	Single	Partnership	p	Not working	Working	p
ADL	NI	32,37±21,28	30,02±17,99	0,640	27,46±16,28	33,36±21,43	0,256	33,85±20,94	29,55±19,27	0,379	26,09±16,15	42,11±22,40	**0,002**
	I	27,00±21,33	36,33±23,38	0,139	30,20±20,28	32,41±25,00	0,728	30,52±22,66	32,04±22,89	0,811	28,29±22,10	37,50±22,79	0,236
	*Ref*	*54,21±27,18*	*54,11±27,09*	*0,936*	*48,95±26,23*	*59,92±27,06*	***<0,001***	*56,13±28,54*	*51,77±26,23*	***0,002***	*41,47±24,55*	*61,31±25,65*	***<0,001***
PWB	NI	46,37±26,03	56,57±20,58	0,093	44,6±24,04	52,84±24,49	0,195	51,28±26,06	49,34±23,42	0,747	51,04±23,67	52,63±25,88	0,813
	I	42,86±25,95	60,76±21,80	**0,010**	50,32±28,42	51,92±22,83	0,824	47,83±27,42	54,17±23,77	0,377	49,54±24,20	59,09±28,28	0,276
	*Ref*	*53,60±24,01*	*61,84±22,01*	***<0,001***	*53,17±24,53*	*59,19±22,08*	***<0,001***	*57,05±25,16*	*54,75±23,28*	*0,069*	*51,50±24,92*	*57,96±23,04*	***<0,001***
RFr	NI	64,53±24,06	59,29±25,96	0,398	66,67±26,48	60,64±23,93	0,349	54,84±28,04	68,86±19,92	**0,018**	64,77±25,66	63,60±20,07	0,860
	I	62,20±22,39	58,33±24,94	0,558	55,77±24,24	65,06±22,11	0,155	55,67±26,76	64,81±19,38	0,162	63,19±23,18	54,55±26,18	0,299
	Ref	65,36±25,04	59,24±26,40	**<0,001**	62,04±25,50	66,89±24,03	**<0,001**	61,98±26,80	64,46±24,26	0,074	63,63±25,11	64,05±25,15	0,754
SPT	NI	56,83±23,46	62,02±21,28	0,360	46,02±22,37	64,76±20,37	**0,001**	56,25±24,15	60,86±21,44	0,405	55,54±23,47	62,50±19,54	0,262
	I	43,97±24,74	56,77±20,43	**0,050**	45,19±23,14	54,57±23,42	0,153	49,00±22,80	50,69±24,60	0,798	51,04±24,07	46,59±25,37	0,599
	*Ref*	*65,45±23,97*	*69,88±21,66*	***<0,001***	*63,56±23,78*	*70,30±22,81*	***<0,001***	*67,97±23,94*	*65,09±23,38*	***0,021***	*60,10±24,48*	*70,04±22,24*	***<0,001***
RFa	NI	72,48±26,32	71,15±23,24	0,833	64,39±32,85	75,53±19,84	0,085	64,25±26,89	78,29±21,79	0,019	69,89±24,59	79,82±24,27	0,145
	I	69,35±26,75	72,57±22,05	0,641	74,04±26,07	67,63±22,89	0,351	63,00±26,69	78,09±20,17	**0,025**	69,68±25,60	75,00±25,55	0,549
	*Ref*	*75,05±23,14*	*75,42±22,90*	*0,748*	*73,79±23,97*	*76,38±21,67*	***0,030***	*69,63±25,68*	*76,83±21,67*	***<0,001***	*74,05±22,87*	*74,96±23,66*	*0,455*
RHCS	NI	70,16±19,01	68,91±20,76	0,800	78,79±19,54	65,43±18,22	**0,007**	65,05±18,44	73,46±19,84	0,075	68,94±20,44	74,56±14,82	0,284
	I	70,83±17,93	65,63±22,16	0,353	72,76±20,35	64,10±18,97	0,119	65,33±21,07	71,30±18,82	0,286	68,52±18,91	65,91±25,67	0,715
	*Ref*	*77,69±20,10*	*77,94±20,36*	*0,804*	*77,66±20,21*	*79,78±18,28*	***0,037***	*77,43±21,55*	*78,58±18,87*	*0,276*	*75,76±21,56*	*79,18±18,86*	***0,001***
SSL	NI	50,94±31,19	42,71±32,33	0,317	45,45±35,05	49,11±30,04	0,664	39,81±32,34	53,72±30,17	0,082	46,34±32,38	55,15±33,09	0,353
	I	50,46±35,27	45,45±33,97	0,618	53,13±35,97	43,50±32,90	0,333	36,36±38,36	57,87±27,98	**0,028**	50,76±35,76	48,86±31,35	0,876
	*Ref*	*61,60±31,90*	*59,96±31,78*	*0,342*	*58,30±32,35*	*64,35±30,14*	***0,001***	*50,84±35,92*	*63,50±29,64*	***<0,001***	*57,22±32,73*	*62,62±31,12*	***0,004***
COP	NI	50,87±30,05	56,25±28,78	0,467	50,57±29,75	53,99±29,62	0,657	52,02±33,24	53,62±26,46	0,824	53,13±30,87	59,87±24,85	0,404
	I	50,45±29,16	60,94±30,02	0,208	50,00±28,28	60,58±30,76	0,203	51,50±30,47	58,80±29,17	0,382	55,90±29,65	54,55±28,1	0,894
	*Ref*	*61,82±30,78*	*64,83±29,65*	*0,052*	*57,98±30,97*	*68,10±28,01*	***<0,001***	*61,58±30,98*	*61,89±30,03*	*0,848*	*57,40±31,50*	*64,41±29,44*	***<0,001***
REJ	NI	63,66±32,83	66,83±29,14	0,687	67,05±32,86	63,83±30,87	0,694	65,73±30,44	64,14±32,38	0,836	68,18±30,32	65,13±26,54	0,705
	I	56,70±37,19	78,13±34,03	**0,036**	66,35±36,53	66,83±38,23	0,963	58,50±40,62	74,07±32,32	0,131	66,32±37,42	68,18±38,47	0,886
	*Ref*	*74,88±26,37*	*76,74±25,22*	*0,168*	*72,38±27,48*	*80,42±21,84*	***<0,001***	*75,95±27,16*	*74,82±25,47*	*0,475*	*71,55±27,46*	*78,54±24,39*	***<0,001***
Index	NI	55,88±10,37	58,19±12,13	0,420	54,56±11,60	57,89±10,67	0,254	53,43±10,55	59,16±10,87	**0,039**	56,60±10,88	60,78±9,37	0,172
	I	53,12±12,30	58,66±16,76	0,188	54,19±12,79	56,98±16,26	0,509	49,97±16,00	60,20±11,70	**0,013**	55,64±12,88	56,69±21,32	0,844
	*Ref*	*65,63±14,95*	*66,46±14,28*	*0,337*	*63,22±14,66*	*69,04±14,00*	***<0,001***	*63,36±16,38*	*65,85±14,14*	***0,020***	*61,41±13,96*	*67,76±14,66*	***<0,001***

ADL activity of daily living, PWB psychological well-being, RFr relationships with friends, SPT symptoms, RFa relationships with family, RHCS relationships with health care system, SSL sentimental and sexual life, COP coping, REJ rejection.

NI non-impaired, I impaired, Ref reference population.

Bold values: p<0,05.

Italic characters: reference population values.

## Discussion

While the assessment of quality of life in MS has received increasing recognition as an outcome parameter in MS research, one should consider whether self-reported information remains reliable when patients experience cognitive problems and to what extent HRQoL measurement remains valid in such a context. Therefore, it seems absolutely necessary to check if the initial internal structure of the self-reported measure is well adapted when HRQoL measures will be used for cognitively impaired individuals and to confirm if the psychometric properties are satisfactory in these populations [3].

Our results provide strong arguments to support the conclusion that cognitively impaired MS patients, as defined from an executive dysfunction, are reliable and consistent when answering the MusiQoL questionnaire. First, the confirmatory factor analysis showed that the structure performed among the impaired subjects almost matched with the initial structure of the MusiQoL. Overall, 8 of the 9 dimensions were clearly identified. Items describing the predefined rejection dimension mainly contributed to the psychological well-being dimension. The limitation regarding the relative small size of the sample and the meaning of the items describing this rejection dimension which are not so fairly distant to the items constituting the psychological well-being dimension should be noted. This last point can be supported by the examination of the moderate correlation between the 2 dimensions issued of the initial validation (r = 0.39, p<0.001, data not shown in the initial publication) [19]. However, the unidimensionality of each of these dimensions seemed ensured by the satisfactory INFIT statistics. Moreover, IIC and IDV values reported in the impaired group were very close to those of the reference population, and similar to those of the non-impaired sample. Internal consistency coefficients, despite the patient's cognitive status, were near to the initial reference population, except for the coping dimension (which presented a less satisfactory coefficient). Floor and ceiling effects were similar to those reported in the initial validation publication, except for the floor effect which was higher for sentimental and sexual life in both the impaired and non-impaired groups. This was probably due to the specificity of the French sample, whereas reference values were issued from patients from 14 countries including North-American subjects. Indeed, it is now well-known that the populations of south Europe more easily accept sexuality as a normal part of life than do North-American populations [33]. Lastly, no difference was found for item functioning, whatever the cognitive status, indicating the relevance of the structure.

Otherwise, the MusiQoL scores of both groups were consistent with those of the SF36 as compared to the reference population. As expected, activity of daily living was strongly linked to the ‘physical-like’ dimensions of SF36 (including the physical composite score), and psychological well-being was highly correlated to the ‘psychological-like’ dimensions of SF36 (including the mental composite score). These findings support the validity of the MusiQoL in altered and non-altered patients adding information not covered by the generic questionnaires [34].

However, some limitations should be considered. The sample size was small but similar to other studies [10], [11]. The representativeness of our sample should also be noted. Our patients had a more severe disability profile and a higher proportion of secondary progressive disease as compared to international and European MS populations [19], [35]. However, the proportion of cognitively impaired subjects, 44%, was in accordance with the literature [4], [5] and was similar to other studies with like objectives [10], [11]. Nevertheless, the present study did not focus on the most severe cases because patients with dementia or those unable to be assessed using neuropsychological tests were not included.

Another important aspect of this study regards our definition of cognitive dysfunction. Indeed, cognition can be defined as a mental process of knowing, including aspects such as awareness, perception, reasoning, and judgment. Several theoretical approaches to the definition of cognitive impairment can be implemented [36]. The ‘all or none’ approach is based on existence or absence of an abnormality. Another approach is an epidemiologically (or ‘categorically’) based approach [37], which determines that cognitive domains affected in MS patients may be similar between individuals. We arbitrarily restricted cognitive function to its composite executive function to produce additional insight as compared to the two main studies reporting similar data, which focused on memory assessment. Cognitive impairment was defined from the Symbol Digit Modalities Test (SDMT) [11] and from both the Wechsler Adult Intelligence Scale (WAIS-III) and the Wechsler Memory Scale (WMS-III) [10]. Considering just one composite would not have been a perfect reflection of a global cognitive function. It would have been misleading to assume that our patients were not suffering from other neuropsychological deficits [38]. It has been well documented in previous studies that it would be unusual to observe executive deficits in isolation [39], [40] and that HRQoL measurement may be altered differently depending on the kind of cognitive impairment in patients [41]. Executive dysfunction is a frequent finding in MS patients, even at the early stage of the disease. On a psychometric point of view, a recent meta-analysis reported than Stroop word and color test was a more sensitive task to detect executive dysfunction in MS [42]. Future studies could provide further information according to other definitions of cognitive dysfunction integrating combination of different composites (i.e., memory, attention, and concentration).

In the same way as defining executive dysfunction from one test, the Stroop test can also be biased. Because the test requires the use of different cognitive functions such as memory, concentration and executive functions, individuals with different incapacities can be categorized in the same group although they do not present the same deficit [43]. First, while this test is recognized as a good performance tool to assess inhibition ability, general speed of processing [27], and attention performance [44], executive function can include other components such as working memory, initiation and inhibition of responses, strategy planning and conceptual activity, which are insufficiently assessed by the Stroop test. Second, while this test is considered as a standardized neuropsychological instrument, several procedural variations and performance reports are available leading to various interpretations [45], [46]. Our choice to implement this test relied on the following points: i) the high sensitivity of the test [47]; ii) the recent availability of French norms, taking account age and educational level effects [48], eliminating the need for a control group [49]; iii) the existing relationships between Stroop performance and QoL [43]; iv) the frequency of impairment of the Stroop performance in MS population [50]; and v) the cultural robustness, including a French language version [51].

Our study confirms preliminary results reported from two similar previous studies using different QoL measurements and suggesting that executive dysfunction did not compromise the reliable and valid assessment of self-reported health measures. These robust results will be confirmed by performing other cognitive composites, such as memory or attention, among more severely affected individuals. If these findings will be confirmed, assessment of QoL in MS patients could be more widely used without fear of inadequacy of this approach in those patients with cognitive impairment.

## Supporting Information

Table S1 **KMO Kaiser-Meyer-Olkin index.** ADL activity of daily living, PWB psychological well-being, RFr relationships with friends, SPT symptoms, RFa relationships with family, RHCS relationships with health care system, SSL sentimental and sexual life, COP coping, REJ rejection. Factor loadings lower than 0.30 are not reported.
(DOC)

Table S2 **ADL activity of daily living, PWB psychological well-being, RFr relationships with friends, SPT symptoms, RFa relationships with family, RHCS relationships with health care system, SSL sentimental and sexual life, COP coping, REJ rejection.** MCS mental composite score, PCS physical composite score. NI non-impaired, I impaired, Ref reference population. Spearman rank correlation coefficients were presented. Bold values: p<0,05, *p-value <0,05, **p-value <0,01. Italic characters: reference population values.
(DOC)
