# Supplementary material for: Is the Concept of Quality of Life Relevant for Multiple Sclerosis Patients with Cognitive Impairment? Preliminary Results of a Cross-Sectional Study
Source: PLoS One. 2012 Jan 23;7(1):e30627. doi: 10.1371/journal.pone.0030627 (PMC3264575; doi:10.1371/journal.pone.0030627)
Supplement: Table S1 — KMO Kaiser-Meyer-Olkin index. ADL activity of daily living, PWB psychological well-being, RFr relationships with friends, SPT symptoms, RFa relationships with family, RHCS relationships with health care system, SSL sentimental and sexual life, COP coping, REJ rejection. Factor loadings lower than 0.30 are not reported. (DOC) [file pone.0030627.s001.doc]

**Table S1**. Construct validity according to the cognitive status

|  | | Non-impaired N=69 | | | | | | | | | Impaired N=55 | | | | | | | | |
| --- | --- | --- | --- | --- | --- | --- | --- | --- | --- | --- | --- | --- | --- | --- | --- | --- | --- | --- | --- |
| **KMO** | | 0.54 | | | | | | | | | 0.56 | | | | | | | | |
| **Dom** | **Items** | 1 | 2 | 3 | 4 | 5 | 6 | 7 | 8 | 9 | 1 | 2 | 3 | 4 | 5 | 6 | 7 | 8 | 9 |
| **ADL** | 1 | 0,854 |  |  |  |  |  |  |  |  |  | 0,767 |  | 0,321 |  |  |  |  |  |
|  | 2 |  |  | -0,464 |  | 0,399 |  |  |  | 0,354 |  | 0,464 |  | 0,509 |  |  |  |  |  |
|  | 3 | 0,769 |  |  |  |  |  |  |  |  |  | 0,860 |  |  |  |  |  |  |  |
|  | 4 | 0,860 |  |  |  |  |  |  |  |  |  | 0,711 |  |  |  | 0,358 |  |  |  |
|  | 5 | 0,476 |  |  |  | 0,372 |  |  |  |  |  | 0,474 |  | 0,438 | -0,436 |  |  |  |  |
|  | 6 | 0,353 |  | -0,387 |  |  |  |  | 0,497 |  |  | 0,386 |  |  |  | 0,459 |  |  |  |
|  | 7 | 0,504 |  |  |  |  |  |  |  |  |  | 0,357 |  | 0,595 |  | 0,363 |  | 0,331 |  |
|  | 8 |  | 0,443 |  |  | 0,375 |  |  |  |  |  | 0,381 |  | 0,612 |  | 0,404 |  |  |  |
| **PWB** | 9 |  |  |  |  | 0,417 |  | 0,476 | 0,339 |  | 0,726 |  |  |  |  |  |  | 0,325 |  |
|  | 10 |  | 0,577 |  |  |  |  | 0,350 | 0,432 |  | 0,891 |  |  |  |  |  |  |  |  |
|  | 11 |  | 0,759 |  |  |  |  |  |  |  | 0,789 |  |  |  |  |  |  |  |  |
|  | 12 |  | 0,700 |  |  |  |  |  |  |  | 0,851 |  |  |  |  |  |  |  |  |
| **SPT** | 13 |  | 0,480 |  |  | 0,466 |  |  |  |  |  |  |  |  |  |  |  | 0,814 |  |
|  | 14 |  | 0,499 |  |  | 0,627 |  |  |  |  |  |  |  |  |  |  |  | 0,716 |  |
|  | 15 |  |  |  |  | 0,749 |  |  |  |  |  |  |  | 0,856 |  |  |  |  |  |
|  | 16 |  |  |  |  | 0,767 |  |  |  |  |  |  |  |  |  |  |  | 0,547 |  |
| **RFr** | 17 |  |  |  | 0,831 |  |  |  |  |  |  |  | 0,719 |  | 0,405 |  |  |  |  |
|  | 18 |  |  |  | 0,869 |  |  |  |  |  |  |  | 0,885 |  |  |  |  |  |  |
|  | 19 |  |  |  | 0,817 |  |  |  |  |  |  |  | 0,906 |  |  |  |  |  |  |
| **RFa** | 20 |  |  | 0,806 |  |  |  |  |  |  | 0,340 |  |  | -0,306 | 0,710 |  |  |  |  |
|  | 21 |  |  | 0,747 |  |  |  |  |  |  |  |  |  |  | 0,813 |  |  |  |  |
|  | 22 |  |  | 0,794 |  |  |  |  |  |  |  |  |  |  | 0,689 |  |  |  | 0,343 |
| **RHCS** | 29 |  |  |  |  |  | 0,878 |  |  |  |  |  |  |  |  |  | 0,794 |  |  |
|  | 30 |  |  |  |  |  | 0,820 |  |  |  |  |  |  |  |  |  | 0,870 |  |  |
|  | 31 |  | -0,325 |  |  |  | 0,648 |  |  |  |  |  | 0,325 |  |  | -0,443 | 0,526 |  |  |
| **SSL** | 23 |  |  |  |  |  |  |  |  | 0,804 |  |  |  |  |  |  |  |  | 0,888 |
|  | 24 |  |  |  |  |  |  |  |  | 0,857 |  |  |  |  |  |  |  |  | 0,886 |
| **COP** | 25 |  |  |  |  |  |  |  | 0,752 |  |  |  |  |  |  | 0,646 | 0,339 |  |  |
|  | 26 |  |  |  |  |  |  |  | 0,759 |  |  |  |  |  |  | 0,839 |  |  |  |
| **REJ** | 28 |  |  |  |  |  |  | 0,800 |  |  | 0,734 |  |  |  |  | 0,340 |  |  |  |
|  | 29 |  |  |  |  |  |  | 0,848 |  |  | 0,745 |  |  |  |  |  |  |  |  |
