# Supplementary material for: Is the Concept of Quality of Life Relevant for Multiple Sclerosis Patients with Cognitive Impairment? Preliminary Results of a Cross-Sectional Study
Source: PLoS One. 2012 Jan 23;7(1):e30627. doi: 10.1371/journal.pone.0030627 (PMC3264575; doi:10.1371/journal.pone.0030627)
Supplement: Table S2 — ADL activity of daily living, PWB psychological well-being, RFr relationships with friends, SPT symptoms, RFa relationships with family, RHCS relationships with health care system, SSL sentimental and sexual life, COP coping, REJ rejection. MCS mental composite score, PCS physical composite score. NI non-impaired, I impaired, Ref reference population. Spearman rank correlation coefficients were presented. Bold values: p<0,05, *p-value <0,05, **p-value <0,01. Italic characters: reference population values. (DOC) [file pone.0030627.s002.doc]

**Table S2. Correlations between MusiQoL and SF36 scores according to the cognitive status**

|  | | ADL | PWB | RFr | SPT | RFa | | RHCS | SSL | COP | REJ | index |
| --- | --- | --- | --- | --- | --- | --- | --- | --- | --- | --- | --- | --- |
| SF36 |  |  |  |  |  | |  |  |  |  |  |  |
| Physical functioning | NI | **0,56**** | -0,04 | -0,10 | -0,01 | 0,03 | | 0,21 | 0,08 | 0,21 | -0,03 | 0,13 |
|  | I | **0,43**** | **0,37*** | -0,09 | -0,08 | -0,07 | | 0,09 | 0,10 | 0,00 | 0,05 | 0,22 |
|  | *Ref* | ***0,78***** | ***0,15***** | ***0,09***** | ***0,29***** | ***0,05**** | | ***0,19***** | ***0,25***** | ***0,21***** | ***0,34***** | ***0,47***** |
| Social functioning | NI | **0,32*** | 0,24 | -0,04 | **0,27*** | 0,11 | | 0,04 | 0,22 | **0,32*** | 0,03 | **0,29*** |
|  | I | **0,30*** | **0,30*** | 0,26 | 0,27 | 0,13 | | 0,26 | 0,04 | 0,25 | **0,52**** | **0,43**** |
|  | *Ref* | ***0,56***** | ***0,39***** | ***0,22***** | ***0,40***** | ***0,20***** | | ***0,23***** | ***0,30***** | ***0,33***** | ***0,37***** | ***0,59***** |
| Role physical | NI | **0,49**** | 0,05 | -0,23 | 0,15 | **-0,29*** | | -0,03 | 0,01 | 0,24 | 0,17 | 0,09 |
|  | I | 0,22 | 0,21 | 0,21 | 0,12 | -0,01 | | 0,21 | -0,15 | 0,17 | 0,26 | 0,19 |
|  | *Ref* | ***0,63***** | ***0,31***** | ***0,12***** | ***0,44***** | ***0,13***** | | ***0,19***** | ***0,22***** | ***0,28***** | ***0,32***** | ***0,52***** |
| Role emotional | NI | **0,35**** | **0,33**** | -0,21 | **0,44**** | -0,03 | | -0,22 | **0,28*** | 0,18 | 0,19 | **0,31*** |
|  | I | 0,04 | **0,35*** | **0,45**** | 0,23 | 0,25 | | -0,04 | 0,17 | 0,16 | 0,16 | **0,44**** |
|  | *Ref* | ***0,47***** | ***0,39***** | ***0,12***** | ***0,43***** | ***0,13***** | | ***0,15***** | ***0,23***** | ***0,34***** | ***0,34***** | ***0,50***** |
| Mental health | NI | **0,30*** | **0,72**** | -0,05 | **0,29*** | 0,06 | | 0,10 | 0,14 | **0,42**** | 0,05 | **0,50**** |
|  | I | **0,30*** | **0,67**** | **0,32*** | 0,17 | **0,40**** | | 0,10 | **0,40**** | 0,26 | **0,44**** | **0,65**** |
|  | *Ref* | ***0,36***** | ***0,65***** | ***0,22***** | ***0,38***** | ***0,29***** | | ***0,22***** | ***0,37***** | ***0,44***** | ***0,41***** | ***0,65***** |
| Vitality | NI | **0,48**** | 0,18 | -0,12 | **0,30*** | -0,21 | | 0,21 | -0,01 | 0,15 | -0,10 | 0,14 |
|  | I | **0,53**** | **0,38**** | -0,15 | 0,24 | -0,13 | | 0,13 | 0,16 | 0,09 | **0,31*** | **0,31*** |
|  | *Ref* | ***0,63***** | ***0,48***** | ***0,19***** | ***0,46***** | ***0,21***** | | ***0,23***** | ***0,33***** | ***0,33***** | ***0,36***** | ***0,62***** |
| Bodily pain | NI | **0,27*** | **0,25*** | -0,12 | **0,33**** | **-0,25*** | | -0,12 | 0,04 | 0,04 | 0,12 | 0,15 |
|  | I | **0,46**** | **0,42**** | -0,14 | **0,28*** | -0,14 | | 0,12 | -0,10 | 0,10 | **0,29*** | **0,34*** |
|  | *Ref* | ***0,46***** | ***0,31***** | ***0,07***** | ***0,44***** | ***0,09***** | | ***0,17***** | ***0,18***** | ***0,21***** | ***0,27***** | ***0,42***** |
| General health | NI | **0,54**** | 0,07 | -0,08 | 0,20 | -0,04 | | 0,02 | 0,21 | 0,25 | -0,14 | 0,13 |
|  | I | **0,43**** | **0,35*** | -0,02 | 0,19 | -0,05 | | 0,12 | 0,13 | 0,23 | 0,21 | **0,37*** |
|  | *Ref* | ***0,50***** | ***0,35***** | ***0,18***** | ***0,33***** | ***0,15***** | | ***0,21***** | ***0,27***** | ***0,36***** | ***0,34***** | ***0,51***** |
| MCS | NI | **0,28*** | **0,60**** | -0,09 | **0,43**** | 0,06 | | -0,02 | 0,22 | **0,39**** | 0,05 | **0,43**** |
|  | I | 0,26 | **0,57**** | **0,48**** | **0,32*** | **0,36*** | | 0,11 | 0,33* | **0,30*** | **0,52**** | **0,68**** |
|  | *Ref* | ***0,30***** | ***0,60***** | ***0,22***** | ***0,41***** | ***0,26***** | | ***0,18***** | ***0,32***** | ***0,41***** | ***0,35***** | ***0,58***** |
| PCS | NI | **0,61**** | -0,17 | -0,23 | 0,09 | **-0,25*** | | 0,09 | 0,04 | 0,08 | -0,00 | -0,00 |
|  | I | **0,47**** | 0,24 | -0,14 | 0,18 | **-0,31*** | | 0,07 | -0,18 | 0,06 | 0,16 | 0,11 |
|  | *Ref* | ***0,77***** | ***0,16***** | ***0,09***** | ***0,37***** | ***0,07***** | | ***0,21***** | ***0,22***** | ***0,21***** | ***0,33***** | ***0,46***** |
